# Single-cell RNA-seq highlights a specific carcinoembryonic cluster in ovarian cancer

**DOI:** 10.1038/s41419-021-04358-4

**Published:** 2021-11-13

**Authors:** Hongyu Zhao, Yan Gao, Jinwei Miao, Suwen Chen, Jie Li, Zhefeng Li, Chenghong Yin, Wentao Yue

**Affiliations:** 1 Central Laboratory, Beijing Obstetrics and Gynecology Hospital, Capital Medical University, 100026 Beijing, China; 2 Department of Gynecologic Oncology, Beijing Obstetrics and Gynecology Hospital, Capital Medical University, 100026 Beijing, China; 3 Department of Family Planning, Beijing Obstetrics and Gynecology Hospital, Capital Medical University, 100026 Beijing, China

**Keywords:** Cancer genomics, Tumour immunology

## Abstract

Expounding the heterogeneity for ovarian cancer (OC) with the cognition in developmental biology might be helpful to search for robust prognostic markers and effective treatments. In the present study, we employed single-cell RNA-seq with ovarian cancers, normal ovary, and embryo tissue to explore their heterogeneity. Then the differentiation process of clusters was explored; the pivotal cluster and markers were identified. Furthermore, the consensus clustering algorithm was used to explore the different clinical phenotypes in OC. At last, a prognostic model was construct and used to assess the prognosis for OCs. As a result, eight diverse clusters were identified, and the similarity existed in some clusters between embryo and tumours based on their gene expression. Meaningfully, a subtype of malignant epithelial cluster, PEG10^+^ EME, was associated with poor survival and was an intermediate stage of embryo to tumour. PEG10 was a CSC marker and might influence CSC self-renewal and promote cisplatin resistance via NOTCH pathway. Utilising specific gene profiles of PEG10^+^ EME based on public data sets, four phenotypes with different survival and clinical response to anti-PD-1/PD-L1 immunotherapy were identified. These insights allowed for the investigation of single-cell transcriptome of OCs and embryo, which advanced our current understanding of OC pathogenesis and resulted in promising therapeutic strategies.

## Introduction

Incidences of ovarian cancer (OC), one of the most fatal and aggressive tumours of the female reproductive system, have increased in recent years [1]. OC patients often face poor prognoses, presumably because of genetic heterogeneity limiting reproducible prognostic classifications.

At present, studies have explored the mechanism of carcinogenesis and developed effective diagnostic methods focussed on the gene expression patterns between OCs and normal samples using bulk transcription [2, 3]. The gene expression profiles have been reported to correlate with overall survival [4–6] and response to platinum therapy [7, 8]. However, extensive heterogeneity exists in OC cells, which is a key mechanism for overall survival (OS) and progression of cancers [9, 10]. Actionable diagnostic markers and therapeutic targets identified based on bulk profiling technologies disregarding intra-tumoural heterogeneity have been controversial and not suitable for all patients. The emerging single-cell technology has provided powerful tools for exploring genetic and functional heterogeneity, thus helping to solve the problem. This technology has been increasingly used in more regions [11–18] and has provided novel mechanisms in our understanding of both carcinogenesis and in revealing strategies for treatment. However, few studies have explored OC at the single-cell level. One recent single-cell RNA-sequencing (scRNA-seq) study [19] investigated tumour heterogeneity at cellular resolution with OC samples. Another study examined how fallopian tube epithelium cells of origin could enable accurate prediction of cancer behaviour [14]. These studies provide novel insights to explore the carcinogenesis of OC, and their findings have enhanced our understanding of OC.

In the 1970s, Pierce et al. [20] presented a theory that cancer is a problem of developmental biology, and the embryo may control the process. With the increased cognition in developmental biology, researchers have discovered the similarity of biological behaviours between early embryo development and tumorigenesis, as well as the important interaction between tumours and embryos [20–23]. Studies have demonstrated that the similarity exists between embryo development and tumorigenesis at the level of gene and protein expression and their important biological behaviours [22–26]. It was necessary to explore the heterogeneity of OCs and embryos and the underlying clues that are crucial for OC diagnostic/therapeutic strategies. To our knowledge, this is the first study to define in detail a single-cell atlas of the OCs and embryo. Understanding OC progression in the perspective of developmental biology could help for deeply comprehending the mechanism of carcinogenesis, which might provide new insights for anticancer therapy.

In our study, we employed 10× single-cell sequencing to study the heterogeneity of OCs and embryo and explored pivotally prognostic phenotypes or markers in regulating OC progression. We found the that embryo was similar with OCs in several clusters because of similar gene expression patterns. Moreover, we identified PEG10^+^ embryonic malignant epithelial (EME) cell as a carcinoembryonic cluster, which was associated with poor survival. Significantly, paternally expressed gene 10 (PEG10) might influence cancer stem cell (CSC) self-renewal and promote cisplatin resistance via NOTCH pathway. We further explored the clinical application of novel genes of PEG10^+^ EME with public data sets. Thus, our findings provide unprecedented insights for understanding OC progression and establish four distinct immune phenotypes with different OS.

## Results

### Single-cell expression atlas of OC tumours and embryo tissue

**Fig. 1:**
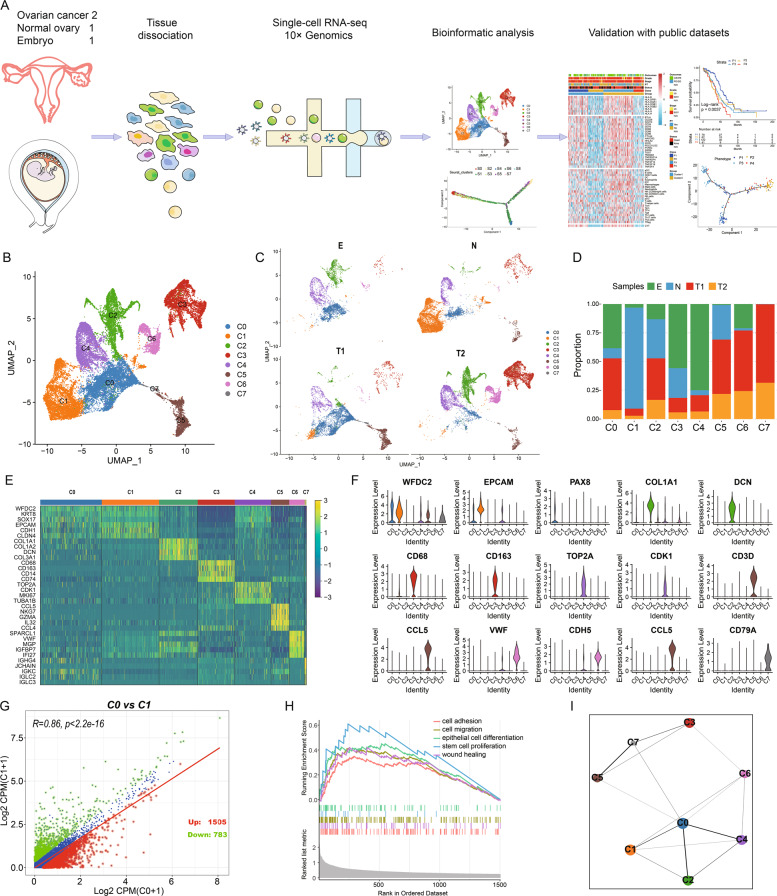
Integrated analysis of OCs and embryonic single-cell transcriptomes. **A** Workflow illustrating collection and processing of single-cell transcriptome of OCs, control ovary, and embryo. **B**, **C** OCs and embryo cell clusters from 10× Genomics scRNA-seq analysis visualised by UMAP. **D** The proportion of diverse cell types across different samples. **E** Heatmap displaying the expression patterns of specific markers in each cell type. **F** Violin plots exhibiting the expression of representative markers across diverse cell types. **G** Scatter plots showing the gene expression patterns of type C0 (*x* axis) and C1 (*y* axis). **H** GSEA analysis of C0 displaying its’ malignant characteristics. **I** Relationship of C0 and other cell types.

To explore the cellular diversity in OC and embryo tissue, two OC samples and one normal ovary were taken from two patients, and ninth-week embryo tissue was obtained from an aborted foetus that a previously pregnant woman donated (Fig. 1A). After removing low-quality cells, we acquired single-cell transcriptomes in a total of 16,027 cells from OC samples, 7655 cells from the control ovary, and 3628 cells from embryonic tissue. After removing batch effects among multiple samples, we identified eight main clusters (Fig. 1B, C). Based on the expression of well-known markers (Fig. 1E, F), which were strongly and specifically marked regarding each major cell population, we annotated the cells as epithelial clusters (C0 and C1), mesenchyme cluster (C2), macrophage cluster (C3), differentiation cluster (C4), T cell cluster (C5), endothelium cluster (C6), and B cell cluster (C7).

As is known, OC majorly originates from epithelium. Among the eight diverse clusters identified in the present study, two different types of epithelial cells (C0 and C1) encouraged us to investigate their malignant status and seek the potential epithelial cells associated with carcinogenesis. We found that C0 consisted of more proportions of tumour cells and embryo cells. In contrast, C1 illustrated a more normal cell population (Fig. 1C, D). The peculiar marker for clinical identification of the OCs, such as PAX8, was only existent in the C0 (Fig. 1F). In addition, we compared discrepant genes between C0 and C1, identifying 1505 upregulated and 783 downregulated genes in C0 (Fig. 1G). Functions of C0 were focussed on some carcinogenesis terms, such as cell adhesion, cell migration, and epithelial cell differentiation (Fig. 1H). Next, we focussed on the transcriptional correlations between the eight clusters. This revealed that C0 showed the most interaction with other cell types (Fig. 1I).

Using pySCENIC pipeline, we identified the regulons for each cluster. The most specific regulons associated with C0 were ZIC5, ZIC2, POU3F1, PGAM2, and LHX1 (Fig. S1A), which associated with carcinogenesis or embryo development. Strikingly, 378 regulons were organised into 10 major modules (Fig. S1B). For each module, we identified several representative regulators and cell types with their average activity scores (Fig. S1B, C). Significantly, we found that C0 was the most frequent cluster in these modules. This demonstrated that C0 was the pivotally malignant epithelial cluster and exploring this cluster might deepen our understanding of OC progression.

### Comparison of OCs and embryo

**Fig. 2:**
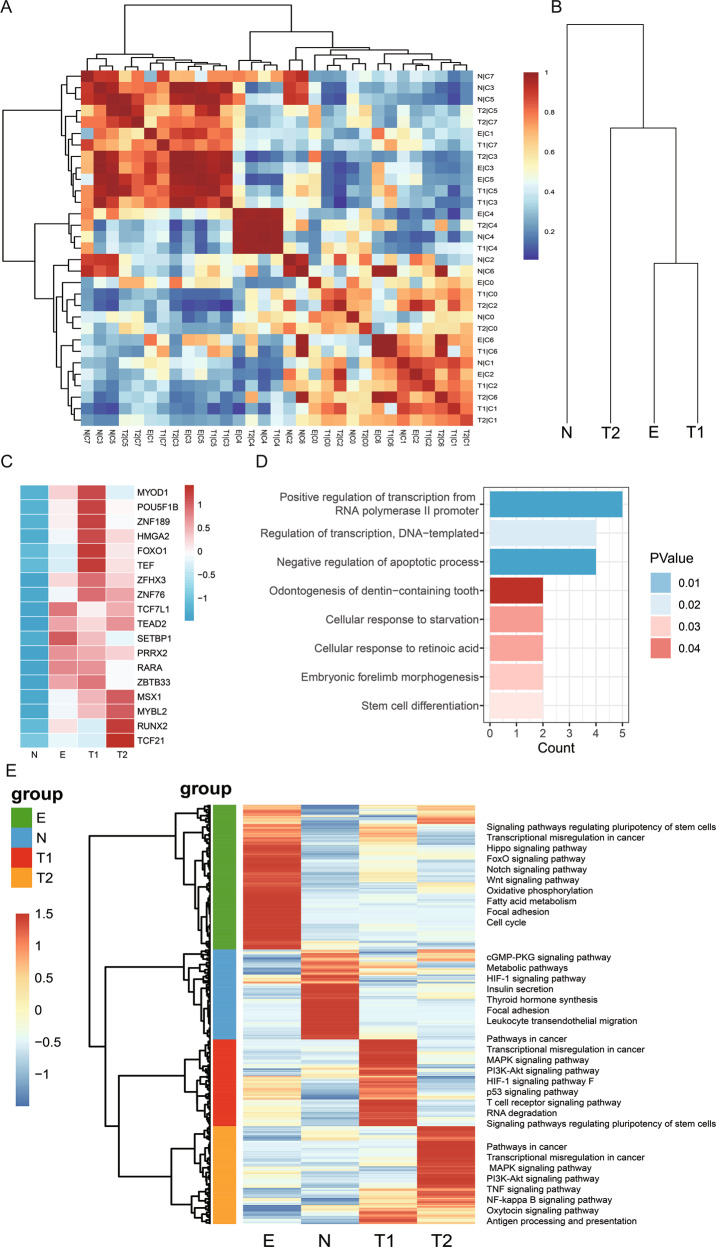
Comparison of diverse clusters and samples. **A** Heatmap showing conserved cell types in OCs, normal ovary, and embryo. **B** Evolutionary tree illustrating the similarity between samples. **C** Heatmap of 18 differential regulons by SCENIC for different samples. **D** Functional analysis of the 18 regulons. **E** Heatmap illustrating diverse genes and relative pathways in four samples.

Studies have stated that similarity existed between embryo development and tumorigenesis based on bulk data or epigenetics data [23–26]. In the present study, we investigated the similarity between tumours and embryonic tissue at single-cell levels. A heatmap indicated that clusters in tumours were similar to those in embryo tissue (Fig. 2A), which was validated using the evolutionary tree (Fig. 2B).

In addition, we discovered that several regulons were activated in the embryo and tumours but deactivated in the normal ovary (Fig. 2C). They were enriched in the functions of embryo development, such as regulation of transcription and stem cell differentiation (Fig. 2D). This suggested that embryo development was similar to tumorigenesis. However, some genes were discrepant and dynamic between tumours and embryo tissue that decided their different destiny. In this study, we also explored several differentially expressed genes (DEGs) as well as several pathways between the four samples (Fig. 2E). These results increased our understanding of cancer progression.

### A distinct carcinoembryonic subgroup identified in epithelial cluster

**Fig. 3:**
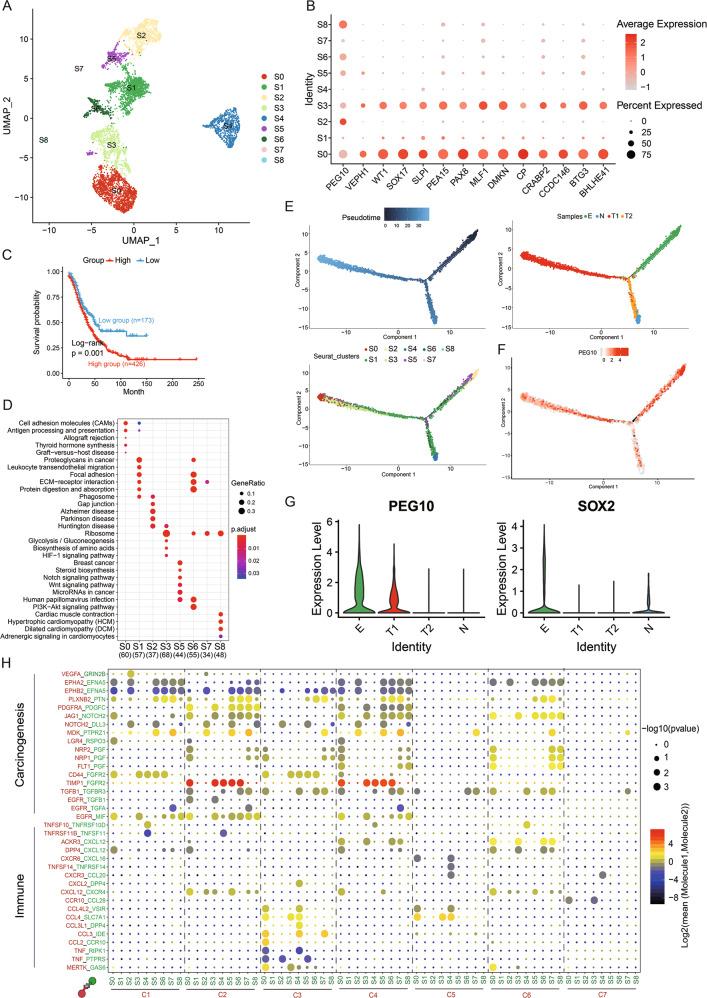
Differential gene expression profiles of diverse clusters of C0. **A** UMAP representation of nine subgroups generated from C0. **B** Dotplot illustrating the representative genes across diverse cell types. **C** Kaplan–Meier curves for patients with high- and low-GSVA score (based on top 100 markers of S3) in GEO OC meta data sets (Log-rank *p* = 0.0099). **D** Functional analysis of each subtype is illustrated with KEGG. **E** Pseudotime of nine subtypes and four samples in C0 inferred by Monocle2. Each point corresponds to a single cell. Clusters or samples information are shown. **F** Expression of PEG10 was mapped to the single-cell trajectory plot. Colour from grey to red indicates relative expression levels from low to high. **G** Violin plots illustrating the mRNA levels of PEG10 and SOX2 in samples based on cells in S3. **H** Heatmap of selected ligand–receptor interactions between diverse cell types and subtypes of C0. Point size indicates *p* value (CellPhoneDB). Colour indicates the mean expression level of ligand and receptor (Mol1/2).

To identify the heterogeneity of epithelial cells in OC, we reclustered the malignant epithelial cells and identified nine distinct subgroups based on UMAP analysis (Fig. 3A). We noticed that both S3 and S0 cells expressed several malignant genes for identification in the OCs (Figs. 3B and S2A). A higher gene set variation analysis (GSVA) score for S3 explicated poor OS in the OC Gene Expression Omnibus (GEO) data sets (Fig. 3C). Functions of S3 were associated with ribosomes and the hypoxia-inducible factor 1 (HIF-1) signalling pathway (Figs. 3D and S2B). The pseudotime graph demonstrated that a differentiation process existed from embryo to tumour (Figs. 3E and S2C), and the heatmap illustrated the dynamic gene expression and related pathways from embryo to tumour (Fig. S2D). Significantly, S3 was the intermediate state of the embryo-to-tumour process (Figs. 3E and S2C). This demonstrated that S3 played an important role in the transformation from embryo to tumour. In our study, PEG10 was a marker gene of S3, which was mainly expressed in embryo and malignant tumour (Figs. 3B, G and S3B). With the development of embryo, the expression of PEG10 was lessened but ascended in the tumour (Fig. 3F), thus highlighting its important role in the early period of embryo to tumour. Thus, we named S3 as PEG10^+^ EME cells.

To explore the molecular interaction networks between PEG10^+^ EME and other cell types, CellPhoneDB was used to analyse the seven major cell types and nine cell subclusters of C0. The heatmap indicated that S3 was related to immunity and carcinogenesis (Fig. 3H). The immune molecules—CXCR4, CCL28, SLC7A1, and CXCL12—secreted by S3 cells interact with receptors expressed on C2–C7. In addition, S3 cells also secreted carcinogenesis molecules—FGFR2, MIF, TGFB1, PDGFC, and NOTCH2—that interacted with JAG1, VEGFA, EGFR, PDGFRA, and TGFB1, which were expressed in C1–C7. Similar ligand–receptor pairs were also found between S2, S7, S8 (which majorly consist of embryonic cells), and C1–C7. This strengthened the assertion that tumorigenesis was similar with embryo development.

### PEG10 deficiency inhibiting stem cell self-renewal and promoting cisplatin-resistance

**Fig. 4:**
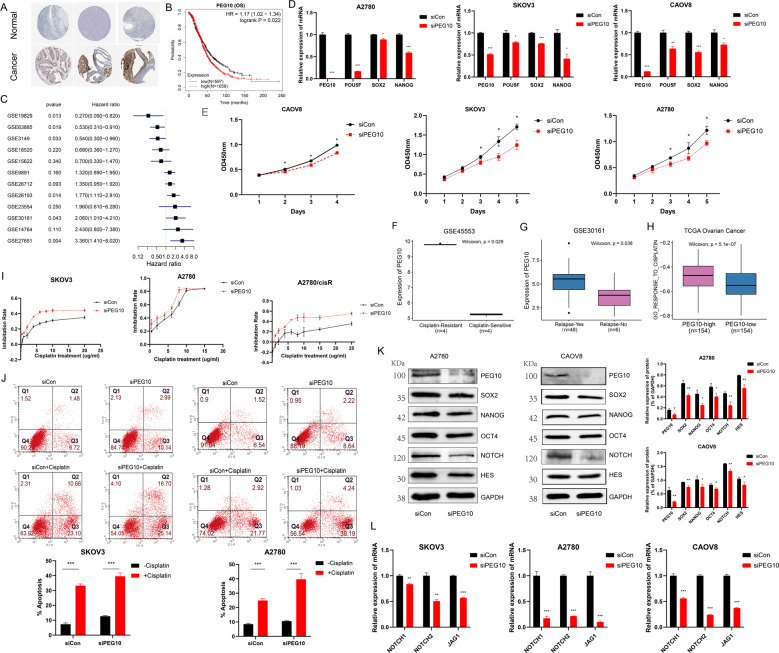
PEG10 deficiency inhibits tumour cell proliferation and promotes cisplatin resistance by targeting stem cell self-renewal. **A** Representative protein expression levels of PEG10 were high in OCs based on the Human Protein Atlas database. **B** Patients with higher PEG10 illustrated poor OS based on Kaplan–Meier plotter data set. The cut-off of PEG10 was 1038 to dichotomise OC patients. **C** Forest plot showing PEG10 is associated with poor OS in most OC data sets. **D** Barplots demonstrating mRNA expression of SOX2, NANOG, and POU5F were lower in siPEG10 cells. **E** Downregulation of PEG10 inhibited OC cell proliferation. **F**, **G** Boxplot illustrating higher PEG10 in cisplatin resistant (**F**) and relapse (**G**) group based on GEO data sets. **H** GSVA of GO_RESPONSE_TO_CISPLATIN in the high- and low-PEG10 group. **I** Cisplatin sensitivity in siCon and siPEG10 OC cells. **J** Apoptotic cells by flow cytometry in control and siPEG10 OC cells untreated or treated with cisplatin. **K** Protein levels of PEG10, SOX2, NANOG, OCT4, NOTCH, and HES were also lower in siPEG10 OC cells. **L** mRNA levels of NOTCH1, NOTCH2, and JAG1 were lower in the siPEG10 group compared to the siCon group. *represented *p* < 0.05, **represented *p* < 0.01, ***represented *p* < 0.001.

PEG10 is an imprinted gene, which is essential for placental development and plays critical role in mouse embryonic stem cells (ESCs) and trophoblast stem cells (TSCs) [27]. Studies point out that PEG10 promotes carcinogenesis of cancers [28–30]. In the present study, we demonstrated that PEG10 was higher in OC samples (Figs. 4A and S3A). Moreover, patients with higher PEG10 exhibited poor OS (Fig. 4B, C) and progression-free survival (PFS) (Fig. S3C, D). Upon stratification of the samples according to specific clinical features, significant differences in OS (Fig. S3E) and PFS (Fig. S3F) were observed between the low and high PEG10 groups, demonstrating that PEG10 was heterogeneous in different subgroups. SOX2, NANOG, and OCT4 are classical stem markers. Next, we explored the stem characteristic of PEG10. In our study, SOX2 highly expressed in embryonic cells, and PEG10 highly expressed in embryonic and malignant tumour cells (Figs. 3G and S3B). In the scatter plot, PEG10 was positive correlated with SOX2 (Fig. S3G, H). As validation, the mRNA expression of SOX2, NANOG, and POU5F was lower in siPEG10 cells (Fig. 4D). At the protein levels, SOX2, NANOG, and OCT4 were also lower in siPEG10 cells (Fig. 4K). Moreover, downregulation of PEG10 inhibited multiple OC cell line proliferation, such as CAOV8, A2780, and SKOV3 (Fig. 4E). Thus, sufficient evidence demonstrated that PEG10 deficiency had a profound impact on stem cell self-renewal by inhibiting genes involved in pluripotency.

**Fig. 5:**
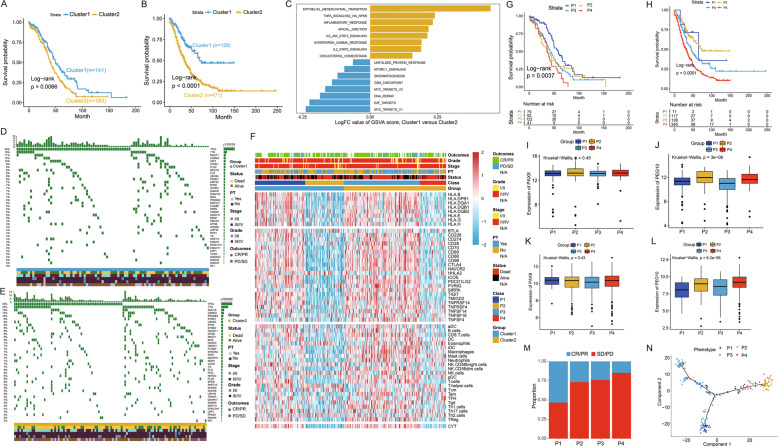
Four phenotypes identified based on markers of carcinoembryonic subtype. **A**, **B** Kaplan–Meier curves for Cluster1 and Cluster2 based on TCGA OC cohort (**A**) and GEO OC meta data sets (**B**). **C** GSVA analysis of two clusters. **D**, **E** Waterfall chart illustration of top 40 mutated genes in the two clusters in OC. A limited number of mutations were in Cluster1 (**D**); more mutations existed in Cluster 2 (**E**). **F** Heatmap representation of immune molecules in four classes in TCGA OC cohort. **G**, **H** Kaplan–Meier curves for patients with four distinct phenotypes in TCGA OC cohort (Log-rank *p* = 0.0037, **G**) and GEO OC meta data sets (Log-rank *p* < 0.0001, **H**). **I**–**L** Boxplot illustrated the expression of PAX8 (**I**, **K**) and PEG10 (**J**, **L**) in four phenotypes based on TCGA OC cohort and GEO OC meta data sets. **M** Ratio of clinical response (CR/PR and PD/SD) to anti-PDL1 immunotherapy in four immune phenotypes based on IMvigor210 cohort. **N** Pseudotime of four phenotypes in TCGA OC cohort inferred by Monocle2. Each point corresponds to one OC sample. Phenotype information is shown.

CSCs are closely related with chemotherapy and radiation therapy resistance. In our study, we found that PEG10 was higher in the cisplatin resistance (Fig. 4F) and relapse group (Fig. 4G). GO_RESPONSE_TO_CISPLATIN was more enriched in PEG10-high group using the GSVA algorithm (Fig. 5H). Then Cell Counting Kit-8 (CCK-8) assay and flow cytometry were performed to discuss the effect of PEG10 on the sensitivity of OC cell lines to cisplatin. SKOV3, A2780, and A2780/cisR cells were transferred with siPEG10 and siCon for 48 h, then the cells were treated with increasing concentrations of cisplatin for 24 h and their inhibition rate was measured by CCK8. As expected, SKOV3 cells transferred with siPEG10 were more sensitive to cisplatin toxicity. Same trend could be found in cisplatin-sensitive and cisplatin-resistant cells, such as A2780 and A2780/cisR (Fig. 4I). Furthermore, downregulation of PEG10 significantly strengthen cisplatin-induced apoptosis (Fig. 4J). These proved that PEG10 deficiency indeed increased cellular sensitivity to chemotherapy.

Further, to determine the molecular mechanism of PEG10 in stem cell self-renewal, GSVA proved that high-PEG10 group was enriched in REACTOME_SIGNALING_BY_NOTCH (Fig. S3I). Convincingly, PEG10 was also positive with markers of NOTCH pathway in cancers (Fig. S3J). Then, reverse transcription–quantitative polymerase chain reaction (RT-qPCR) and western blot were conducted to validate the result. Interesting, protein levels of NOTCH and HES were lower in the siPEG10 group (Fig. 4K); mRNA levels of NOTCH1, NOTCH2, and JAG1 were lower in the siPEG10 group compared to the siCon group (Fig. 4L). Thus, we inferred that PEG10 influenced CSC self-renewal via NOTCH pathway.

### Identification of four phenotypes with different survival utilising a gene panel of 49 markers from PEG10^+^ EME

To elaborate novel subgroups that correlated with survival or treatment response, we first identified 49 of the 901 genes from PEG10^+^ EME with statistical significance utilising the univariable Cox proportional hazards regression analysis based on The Cancer Genome Atlas (TCGA) OC data set (Fig. S4A, B). Then, unsupervised clustering methods (pam) were used, and OC patients were divided into two clusters according to the 49 prognostic genes’ expression (Fig. S4C). Patients in Cluster2 presented poorer survival (Fig. 5A, B) and showed more mutated genes (Fig. 5D, E), demonstrating the malignant status of Cluster2. GSVA analysis demonstrated that Cluster2 was more enriched in the immune-related terms, such as INFLAMMATORY_RESPONSE, IL6_JAK_STAT3_SIGNALING, etc. The other cluster was enriched in E2F_TARGETS, DNA_REPAIR (Fig. 5C). Moreover, patients in Cluster2 were accompanied by greater expression of immune cells, immune checkpoints, and HLAs (Fig. 5A–D).

Immune cytolytic activity (CYT), a measurement for assessing immune infiltration, has been defined as the geometric mean of GZMA and PRF1 expression [31]. In the present study, CYT was associated with multiple immune molecules (Fig. S6), and infiltration of CYT has been shown to be related to better survival in patients with multiple cancers (Fig. S7). This indicated that CYT infiltration could indicate the immunologic dynamics in the tumour microenvironment. Based on the prior cluster and the level of CYT, we divided the OC patients into four phenotypes (P1–P4). Patients in these phenotypes were accompanied by different immune molecules and CYT infiltration (Figs. 5F, S8, and S9A, B), thus these phenotypes might closely correlate with immunotherapy such as immune vaccines and anti-programmed cell death ligand 1 (anti-PD-L1) immunotherapy. Obviously, patients in P1 exhibited better survival, and patients in the P4 class showed worst survival and P2 and P3 had similar survival (Figs. 5G, H and S9C). PAX8 is the malignant clinical marker for OC; however, PAX8 was not helpful for identification of the four phenotypes (Fig. 5I, K). Notably, the CSC marker, PEG10, was lower in P1 and higher in P4 (Fig. 5J, L), demonstrating the malignant status of P4. Thus, the novel CSC marker was better than PAX8 in identification of different immunologic phenotypes. Subsequently, we explored patients’ clinical response to anti-PD-L1 immunotherapy in the four phenotypes. Participants in P1 were associated with therapeutic advantages to anti-PD-1/PD-L1 immunotherapy; in the P4 class, patients had adverse clinical responses. Patients in P2 and P3 had similar clinical responses to anti-PD-1/PD-L1 immunotherapy (Figs. 5M and S9D). The pseudotime of the four phenotypes demonstrated that P1 might be the original and P4 was the terminal. Moreover, P2 and P3 had different differentiation trajectories (Fig. 5N), prompting the two phenotypes to be considered controversial.

Weighted gene co-expression network analysis (WGCNA) was used to identify the special gene modules for each phenotype, and it was found that distinct gene expression patterns existed among the four phenotypes (Fig. S9E–I). Meaningfully, functional analysis demonstrated that P1 was involved in immunologic processes, such as activation of T cells and leucocytes (Fig. S9J); P2 was related to microtubule bundle formation (Fig. S9K); P3 was enriched in epithelial change, such as regulation of *trans*-synaptic signalling and epidermis development (Fig. S9L); and P4 was enriched in extracellular matrix-related processes, such as extracellular structure organisation (Fig. S9M). Considering our former results—that P1 (best survival) was infiltrated with more immune molecules, and P4 (worst survival) was infiltrated with lesser immune molecules—we deemed P1 the immune-active phenotype and P4 the immune-desert phenotype. Thus, the work to elucidate the heterogeneity phenotypes could be crucial in developing therapeutic strategies with better efficacy.

## Discussion

OC has been characterised by poor OS and deficiencies in effective treatment because of a high degree of intra-tumoural heterogeneity. With the increased cognition in developmental biology, researchers have discovered the similarity of biological behaviours between early embryo development and tumorigenesis [23–26, 32]. However, most of these studies conducted with bulk data or epigenetics data have disregarded the heterogeneity of samples. Recently developed scRNA-seq technologies have investigated tumour heterogeneity of OCs [19] and examined how non-cancer cells of origin could predict OC behaviour [14]. To our knowledge, this was the first study to define in detail a single-cell atlas of the OCs and embryo and comprehensively explain the heterogeneity and the underlying mechanism for OC progression.

In our study, eight cell types of OCs and embryo were identified and gene expression profiles in each cell type were elucidated based on scRNA-seq analysis. We found that the embryo was similar to OCs in different clusters because of similar gene expression patterns. Some transcription factors (TFs) associated with embryo development were activated both in embryo and tumours. Studies have found that similarity exists between embryo development and tumorigenesis at the gene and protein expression levels and important biological behaviours [23–26]. Therefore, exploring the mechanisms of carcinogenesis from the perspective of developmental biology was meaningful.

Here malignant epithelium, C0, consisted of more proportions of tumour cells and embryo cells and showed the most interaction with other cell types. Then, C0 was reclustered into nine subtypes, of which, PEG10^+^ EME was the intermediate state of the embryo-to-tumour progression. Functions of PEG10^+^ EME were related with ribosome and the HIF-1 signalling pathway, supporting its embryonic and carcinogenesis characteristics. PEG10 was an imprinted gene, which played critical role in mouse ESCs and TSCs [27]. PEG10 was strongly expressed in the placenta, ovary, and testis. However, PEG10 dysregulation was reported in multiple tumours, such as metastasis prostate tumours [29], hepatocellular carcinoma [33], and endometrial cancer [34]. PEG10 was also closely related to the poor prognosis of cancers [29, 33, 34]. In our study, PEG10 was highly expressed in embryo and tumour and associated with poor survival in OC. Thus, we inferred that PEG10 was a marker of CSCs. CSCs was a small population of highly malignant cells and display many characteristics of ESCs or tissue stem cells. CSCs were responsible for unique characteristics of tumour onset, self-renewal, resistance to radiotherapies and chemotherapies, evasion of immune surveillance, and accelerating recurrence and metastasis [35]. In our study, PEG10 was positively correlated with stem cell self-renewal genes, such as NANOG, SOX2, and OCT4, which was validated with PCR and western blot. Studies pointed out that PEG10 promoted carcinogenesis of cancers [28–30]. Liu et al. [33] demonstrated that TSG101 promotes the proliferation, migration, and invasion of hepatocellular carcinoma cells by regulating the PEG10. Zhang et al. [34] found that IGF2BP1 overexpression stabilises PEG10 mRNA in an m6A-dependent manner and promotes endometrial cancer progression. In our study, we provided direct evidence that PEG10 deficiency inhibited multiple OC cell proliferation and influenced the sensitivity of OC cells to cisplatin by inhibiting CSC self-renewal signalling. CSCs have been implicated in conventional chemotherapy resistance [36]; CSCs were considered the initiators of cancer rebound after successfully combatting tumours. Thus, PEG10 would be a target for cancer treatment and help for personalised anticancer strategies. CSCs often showed continuous activation of highly conserved signalling pathways associated with development and tissue homoeostasis, such as the Wnt, Notch, and Hippo signalling pathways. These pathways were related to CSC self-renewal [37] and have been used to explore new drugs targeting CSCs. In the present study, PEG10 was closely correlated with NOTCH pathway. Therefore, targeting PEG10 might inhibit OC progression and propose a new strategy to treat OC via targeting CSCs.

In our study, significant ligand–receptor pairs related to immunity and carcinogenesis existed in the interactions of PEG10^+^ EME and other cell types. This finding supported the assertion that immune cells and immune molecules played important roles in embryo development and tumorigenesis [21, 24, 38]. Furthermore, tumour progression might result from imbalances between tumour progression and the host’s immune response [39]. Thus, identifying the role of distinct gene expression patterns of PEG10^+^ EME in the immune cell infiltration would contribute to enhance our understanding of antitumour response and guide more effective therapeutic strategies.

Furthermore, we identified four distinct immune-related phenotypes with different survival rates, utilising a gene panel of 49 markers form PEG10^+^ EME. P1 was deemed the immune-active phenotype, in which patients had best OS benefit and showed a high level of immune molecules, whereas P4 was deemed the immune-desert phenotype, in which patients had poorer OS and showed a lower level of immune molecules. Thus, dysregulated gene patterns in PEG10^+^ EME demonstrated a close relationship with diverse immune phenotypes. Moreover, PEG10 was superior than PAX8 to identifying diverse immune phenotypes. In this work, patients with different phenotypes showed distinct response to anti-PD-1/PD-L1 immunotherapy. In P1, patients showed obvious therapeutic advantages to anti-PD-1/PD-L1 immunotherapy; however, patients in P4 had adverse clinical responses. Pseudotime analysis demonstrated that P1 was the original of four phenotypes and P4 was the terminal. Patients in P2 and P3 had different immune molecules infiltration but similar OS and therapeutic advantages to anti-PD1/PDL1 immunotherapy. The two novel, distinct phenotypes demonstrated different differentiation trajectories and were thus controversial. Thus, it is necessary to investigate the two controversial phenotypes in individual tumours. Exploring their gene patterns would help to determine the immune phenotypes of tumours and be informative for exploring mechanisms of immune edition [40, 41], thus guiding more effective clinical practice.

In conclusion, we constructed a single-cell transcriptome atlas of OCs and embryo and provided a new perspective for understanding the progression of OCs. Significantly, we identified a specific carcinoembryonic cluster, namely, PEG10^+^ EME, which played important roles in carcinogenesis. PEG10 influenced CSC self-renewal thus might be a therapeutic target for OCs. Moreover, we identified four distinct phenotypes with different OS based on neoplastic cell-specific marker genes of PEG10^+^ EME. Therefore, a comprehensive assessment of gene patterns of the OCs and embryo would help to enhance not only our current understanding of OC pathogenesis but also the underlying prognosis and treatment for patients.

## Materials and methods

### OC patient and embryo samples

In this study, patients diagnosed with OC or voluntary abortion in the ninth week were enrolled from Beijing Obstetrics and Gynecology Hospital, Capital Medical University in China. The ovarian samples were obtained from the Tissue Bank of Beijing, and the embryonic sample was obtained from the China Birth Cohort. Fresh specimens were collected at the time of surgical resection under the supervision of a qualified pathologist. All the clinical information is listed in Table S1.

### Tissue dissociation and cell purification

Tissues were transported using MACS Tissue Storage Solution (MACS, Cat. no.130-100-008F) on ice to preserve viability. In addition, they were washed 2–3 times with phosphate-buffered saline (PBS; Hyclone, Cat. no. SH30256.01) and then minced on ice. We used the Tumour Dissociation Kit (MACS, Cat. no.130-095-929) to digest the human tissues gently to generate single-cell suspensions. The ovarian tumour and control tissues were dissociated at 37 °C with a shaking speed of 30 rpm for about 6 min. Then we collected the dissociated cells to digest sufficiently with 0.25% trypsin (Gibco, Cat. no.25200056) for about 2 min. The embryonic tissues were then minced and incubated with the same digestion buffer at 37 °C but shaken for about 10 min, then incubated with trypsin for 3 min. Cell suspensions were filtered using a 40 μm nylon cell strainer (Falcon, Cat. no. 352340), and red blood cells were removed. Single-cell suspensions were stained with AO/PI fluorescent dyes (Logos Biosystems, Cat. no. LB F23001) to check viability with LUNA (Logos Biosystems, Cat. no. LUNA-STEM), then diluted with PBS containing 0.02% bovine serum albumin to about 1 × 10^6^ cells/ml for single-cell sequencing.

### scRNA-seq data pre-processing

The concentration of single-cell suspension was 1000 cells/μl. Cells were loaded according to the standard protocol of the Chromium single-cell 3′ reagent kit to capture 5000 cells to 10,000 cells/chip position (V3 chemistry). All the remaining procedures were performed by following the standard manufacturer’s protocol.

Library preparation was performed according to instructions in the 10× Genomics Chromium platform. The libraries were then pooled and sequenced on an Illumina NovaSeq 6000 System. Reads were processed using the Cell Ranger 3.0.1 pipeline (https://support.10xgenomics.com) with default, recommended parameters. FASTQs were generated from Illumina sequencing. The raw sequence files were aligned to the human reference genome (GRCh38) with the STAR algorithm [42]. Finally, a gene–barcode matrix containing the barcoded cells and gene expression counts was generated.

The barcode matrix was processed with Seurat v3 [43], a toolkit for scRNA-seq data analysis. All functions were run using default parameters, unless otherwise specified. Low-quality cells (<300 gene/cells, <3 cells/gene, and >20% mitochondrial genes) were excluded. Then the UMI count data were normalised with log transformation. The highly variable genes (HGVs) were selected to amalgamate samples into a merged data set. Next, the merged cell-by-gene matrix was scaled by dividing the centred expression by the standard deviation.

### Dimension reduction, cell clustering, and annotation

The Seurat package was applied to identify major cell types. HGVs were generated and used to perform graph-based clustering of the principal component (PC) analysis. PCs 1–20 were used for graph-based clustering to identify distinct groups of cells. For sub-clustering, we applied the same procedure of finding variable genes, reducing dimensionality, and clustering to the restricted set of data (usually restricted to one initial cluster). These groups were projected onto UMAP analysis run using the previously computed PCs 1–20.

Next, differential gene expression analysis was performed using the functions of ‘FindMarkers’ or ‘FindAllMarkers’ in Seurat with settings on genes with the default parameters. We characterised the cell types based on classical markers: EPCAM, PAX8, and WFDC2 (epithelium); COL1A1 and DCN (mesenchyme); CD163 and CD68 (macrophage); TOP2A and CDK1 (differentiation cluster); CD3D and CCL5 (T cell); VWF and EMCN (endothelium); CD79A (B cell). The top 100 DEGs in each cluster or subset were then used to perform biological process enrichment analysis.

### Pseudotime analysis

The package Monocle2 [44] was performed to analyse single-cell trajectories or sample trajectories. DEGs over the pseudotime among the different cell clusters or four immune phenotypes’ transitions were calculated by the ‘differentialGeneTest’ function (*q* value <10^−20^ or 10^−2^). ‘DDRTree’ was then applied to reduce dimensions, and functions of ‘plot_cell_trajectory’ were used for visualisation.

### Gene set functional analysis

The gene set functional analysis was conducted with clusterProfiler package [45], gsva package [46], and DAVID (https://david.ncifcrf.gov/home.jsp) [47] with the DEGs in each cluster or subset. The enriched Kyoto Encyclopedia of Genes and Genomes pathways and Gene Ontology terms were derived. H.all.v7.1.symbols.gmt was downloaded from the Molecular Signatures Database (http://www.broad.mit.edu/gsea/msigdb/).

### Construction of a single-cell transcriptome network and cellular similarity analysis

To explore the relationship between the clusters, a toolkit based on python for scRNA-seq data analysis—Scanpy [48]—was used to construct the single-cell transcriptome network with the function of PAGA. To explore the similarity of samples and clusters, the R packages MetaNeighbor [49] and ggtree [50] were utilised.

### SCENIC analysis

TF activity was analysed using SCENIC (v1.0.0.3) for the cell types with raw count matrices as the input. The regulons and TF activity (area under the curve) for each cell were calculated with motif collection version mc9nr according to Suo et al.’s pipeline [51]. Their pipeline included the inference of regulons and their activity, quantifying cell-type specificity score, and regulon module analysis.

### Identification of significant ligand–receptor pairs

To analyse the cell-to-cell communication at the molecular level in our data, we identified significant ligand–receptor pairs using CellPhoneDB [16], a Python-based computational analysis tool. Ligand–receptor pairs with *p* values <0.05 were retained to assess the relationship between the different cell clusters.

### Public data analysis

RNA-seq data and corresponding clinicopathological data of multiple cancer patients in TCGA were obtained from UCSC Xena (https://xenabrowser.net/datapages/). Somatic mutation status for OC (workflow type: Mutec2) was obtained from R package TCGAbiolinks [52]. Data retrieved from multiple GEO databases were used for integrated analysis with R package sva [53]. The IMvigor210 trial data set was extracted from the R package IMvigor210CoreBiologies. All public data used in our study are supplied in Table S2.

Next, unsupervised clustering analysis was used to identify carcinoembryonic patterns and classify patients for further analysis. A consensus clustering algorithm (pam) was then applied to determine the optimal cluster number that was associated with the highest stability and the lowest ambiguity in the TCGA OC data sets. This procedure was performed using the R package ConsensusClusterPlus to ensure the stability of classification.

Marker gene sets for immune cell types were obtained from Gabriela et al. [54] The infiltration levels of immune cell types were quantified by ssGSEA in the R package gsva [46]. CYT was obtained with the mean of GZMA and PRF1.

WGCNA was then performed with the TCGA OC cohort to identify the special gene modules and distinct gene expression patterns for each phenotype with R package WGCNA [55]. A total of 5133 genes in the top 25% of variances were screened for further analysis. In total, 308 samples were analysed, and 11 outliers were eliminated. In the end, 297 samples were used for the analysis, and the soft threshold was 4.

### Cell culture and small interfering RNA (siRNA) transfection

In our study, OC cell lines (including SKOV3, A2780, A2780/cisR, and CAOV8) were obtained from ATCC. SKOV3 and A2780 are cultured in RPMI-1640 medium containing 10% foetal bovine serum (FBS) and 100 U/ml penicillin/streptomycin at 37 °C with 5% CO_2_. A2780/cisR was cultured with the same conditions but the concentration of FBS was 15%. CAOV8 is cultured in Dulbecco’s Modified Eagle Medium supplemented with 10% FBS and 100 U/ml penicillin/streptomycin under similar conditions. All cell lines were transfected using lipofectamine 3000 (Invitrogen, Carlsbad, CA, USA). siRNA against PEG10 was obtained from JTSBIO Co., Ltd. (Wuhan, China). The sequences of siPEG10 were as follows: siRNA1, 5’-CCCAGUGCCAGAUCUUCAUTTAUGAAGAUCUGGCACUGGGTT-3’; siRNA2, 5’-CCAGCUUUCAUGAUGGAAATTUUUCCAUCAUGAAAGCUGGTT-3’, siRNA3, 5’-GCUGGUGUUGCCUCACAUUTTAAUGUGAGGCAACACCAGCTT-3’; and the sequence of siCon was 5’-UUCUCCGAACGUGUCACGUTTACGUGACACGUUCGGAGAATT-3’. Cells were transfected with 100 nmol/l siPEG10 or siCon and incubated for 24 h for subsequent assays.

### Cell proliferation assay

Transfected OC cells were reseeded onto 96-well plates. Then, 10 μl CCK-8 solution (Dojindo, Rockville, MD, USA) was added to each well. After incubation for 2 h, the absorbance of each well was measured at 450 nm using a Tecan Infinite M1000 PRO (Tecan, Switzerland).

### RNA extraction and RT-qPCR

Total RNA was isolated using TRIzol reagent according to the manufacturer’s instructions (Invitrogen, Carlsbad, CA). cDNA was reversely synthesised using the ReverTra Ace qPCR RT Kit (Toyobo, Shanghai, China). The RT-qPCR was performed using the SYBR Premix EX Taq™ (Takala, Dalian, China) in ABI 7500 Real-Time PCR system (Applied Biosystems, Foster City, USA). The primer sequences are listed in Table S3. The expression of glyceraldehyde-3-phosphate dehydrogenase (GAPDH) was used as internal standard. The 2^−ΔΔCT^ comparative method was used to determine the relative gene expression.

### Western blot analysis

Total protein was extracted using RIPA buffer (Thermo Fisher Scientific, Waltham, MA, USA) and measured using BCA assay (Thermo Fisher Scientific, Waltham, MA, USA). Thirty micrograms of protein per sample was separated by sodium dodecyl sulfate–polyacrylamide gel electrophoresis, then transferred onto polyvinylidene fluoride membranes (Gene Molecular Biotech, Inc., Shanghai, China). After blocking with 5% milk for 2 h at room temperature, the membranes were incubated overnight at 4 °C with primary antibodies at the following dilutions: GAPDH (38 kDa, 1:1000, CST), SOX2 (35 kDa, 1:1000, CST), OCT4 (45 kDa, 1:1000, CST), NANOG (42 kDa, 1:1000, CST), NOTCH (120 kDa, 1:1000, CST), HES (30 kDa, 1:1000, CST), PEG10 (100 kDa, 1:1000, Abcam). After that, the membranes were incubated with horseradish peroxidase-conjugated rabbit IgG secondary antibodies (1:7500, CST) for 1 h, and the expression levels were detected by an ECL kit (Roche Diagnostics, Basel, Switzerland) via western blot imaging system.

### Apoptosis assays by flow cytometry

Transfected OC cells were cultured in 6-well plates for 24 h followed by 10 μg/ml cisplatin exposure for 24 h. Subsequently, the cells were collected to determine apoptosis using Annexin-V-fluorescein isothiocyanate and propidium iodide (PI) Kit (BD Biosciences, San Jose, CA, USA). The double stained cells were subsequently analysed with the BD flow cytometer.

All the statistical analyses were performed using the R 3.6.1 and Graphpad prism 8.0 softwares. The assay was repeated at least three times and the data were presented as mean ± standard deviation (SD). The variance was similar between the groups that were being statistically compared. Two-tailed Student’s *t* test was used to assess the differences between two groups. *p* < 0.05 was considered statistically significant.

## Supplementary information

SUPPLEMENTAL MATERIAL

## Data Availability

All the data sets used in the present research are summarised in Table S2. The single-cell raw data have been deposited in the SRA data sets (PRJNA756768).

## Code availability

The code used in our study can be obtained from the corresponding author.
