# Supplementary material for: Single-cell RNA-seq highlights a specific carcinoembryonic cluster in ovarian cancer
Source: Cell Death Dis. 2021 Nov 13;12(11):1082. doi: 10.1038/s41419-021-04358-4 (PMC8590695; doi:10.1038/s41419-021-04358-4)
Supplement: Supplementary file 1 — SUPPLEMENTAL MATERIAL [file 41419_2021_4358_MOESM1_ESM.docx]

**Single-cell RNA-seq highlights a specific carcinoembryonic cluster in ovarian cancer**

Hongyu Zhao^1*^, Yan Gao^1*^, Jinwei Miao^2^, Suwen Chen^3^, Jie Li^1^, Zhefeng Li^1^, Chenghong Yin^1#^, Wentao Yue^1#^

1 Central Laboratory, Beijing Obstetrics and Gynecology Hospital Capital Medical University, Capital Medical University, Beijing 100026, China

2 Department of Gynecology and Oncology, Beijing Obstetrics and Gynecology Hospital Capital Medical University, Capital Medical University, Beijing 100026, China

3 Department of Family Planning, Beijing Obstetrics and Gynecology Hospital Capital Medical University, Capital Medical University, Beijing 100026, China

*These authors contribute equal to this work

^#^ Corresponding authors

E-mail address: yuewt@ccmu.edu.cn (WTY), yinchh@ccmu.edu.cn (CHY)

**Supplementary Figures**

**Fig. S1 Cell-Type-Specific Regulon Activity Analysis**

A Rank for regulons in different clusters based on regulon specificity score (RSS). Blue points represent the top 5 regulons.

B Identification regulon modules based on regulon connection specificity index (CSI) matrix, along with associated cell types, representative transcription factors, and corresponding binding motifs.

C Cell types inferred for each module with their activity scores.


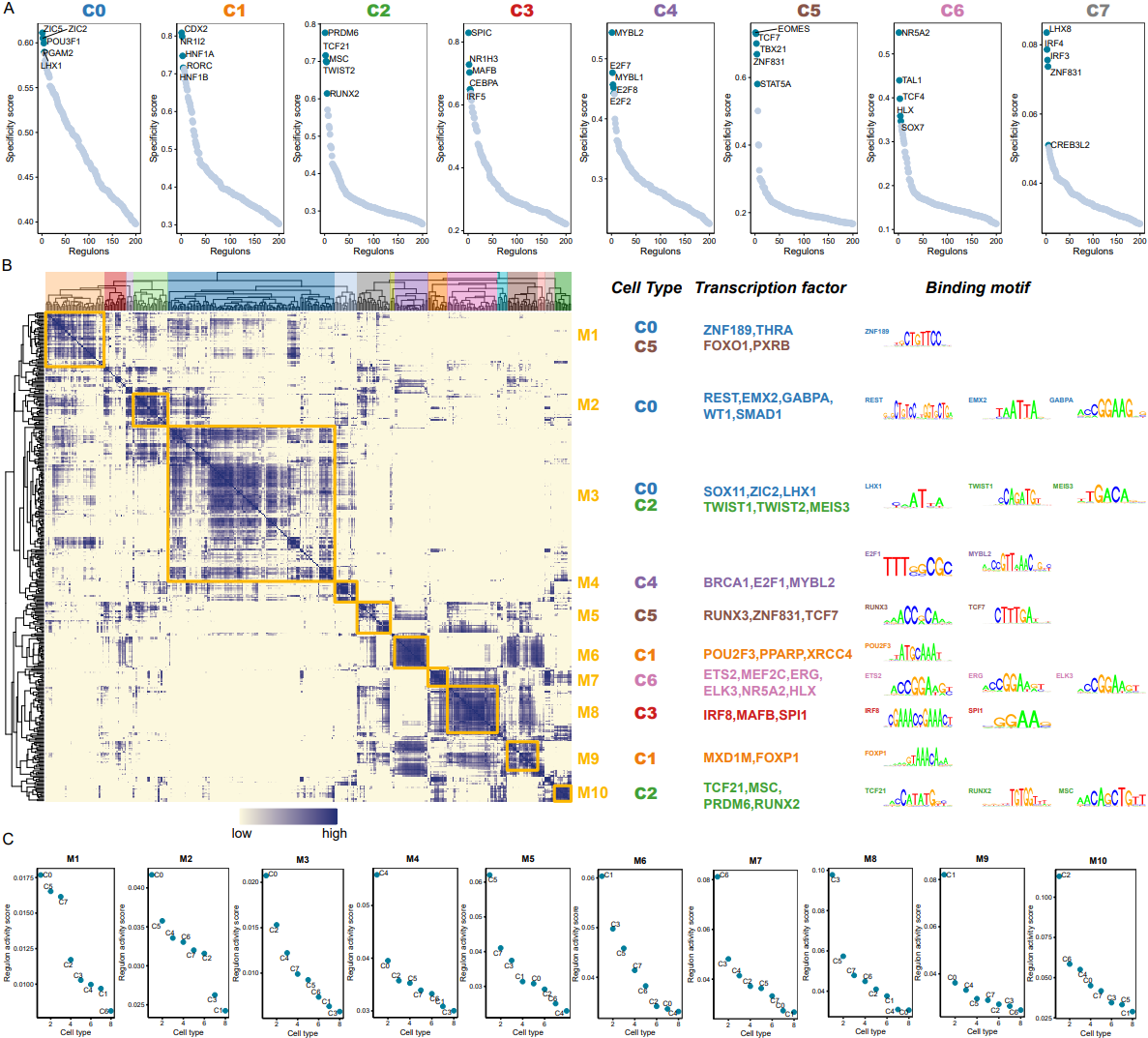


**Fig. S2 Differential gene expression profiles along embryo to tumour.**

A Violin plots displaying the expression of specific markers across diverse cell types.

B Functional analysis of each subtype was illustrated with GO.

C Pseudotime of six subtypes (C) or three samples (D) related to the process of embryo to tumour inferred by Monocle2. Each point corresponds to a single cell. Cluster information is shown.

D Heatmap showing differentially expressed genes rows along the pseudotime with the embryo-to-tumour process. Colour key from blue to red indicates relative expression levels from low to high. Representative gene functions and pathways of each profile are shown.


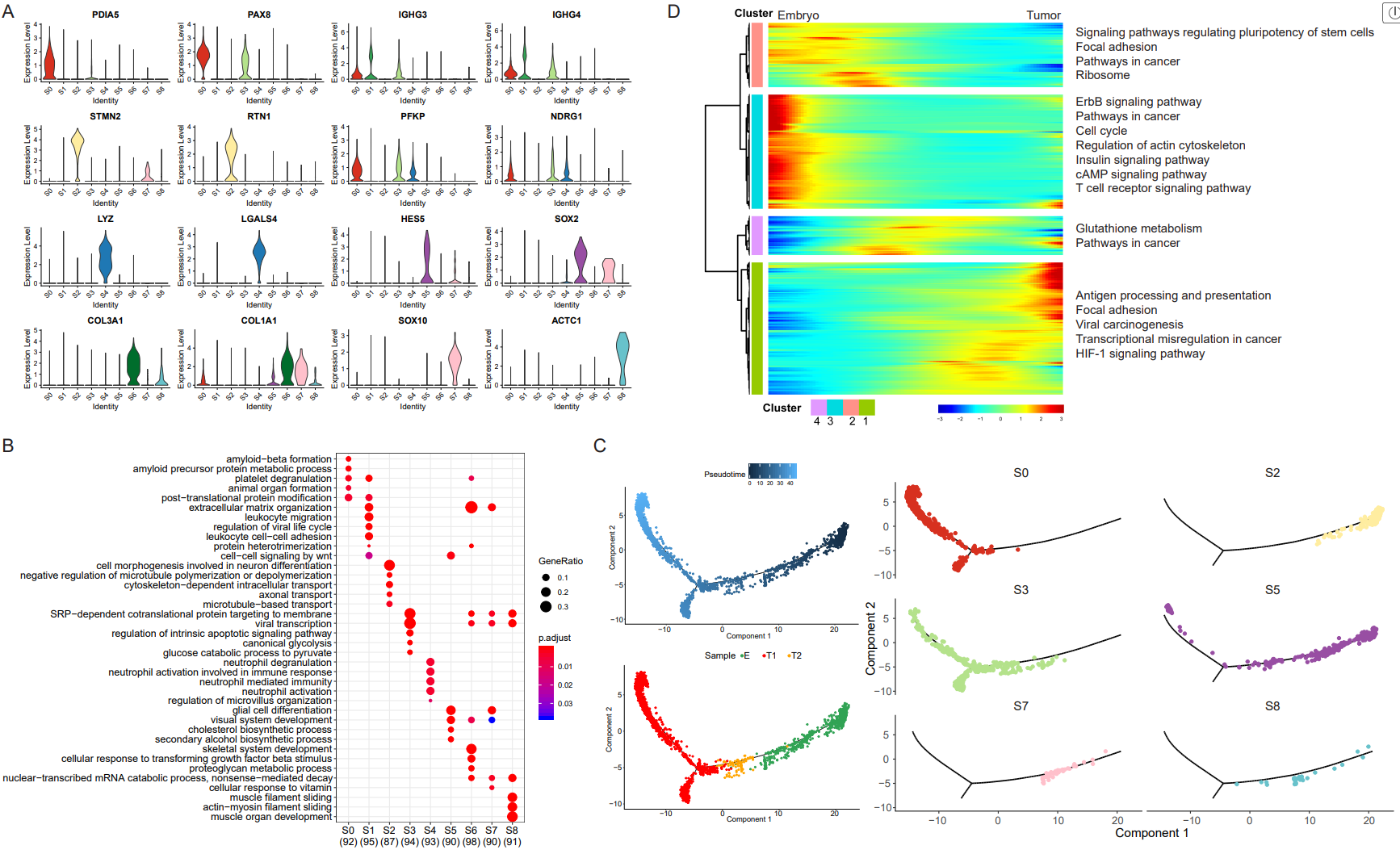


**Fig. S3 Association between PEG10 and survival and stem cell self-renewal.**

A PEG10 was higher in OCs based on Oncomine datasets.

B Violin plots illustrating the mRNA levels of PEG10 and SOX2 in samples based on all cells.

C Patients with lower PEG10 illustrated better PFS based on Kaplan-Meier plotter dataset. The cut-off of PEG10 was 1411 to dichotomize OC patients.

D Forest plot showing PEG10 is associated with poor PFS in most OC datasets.

E, F Forest plots illustrating OS (E) and PFS (F) associated with PEG10 in different subgroups in OC patients.

G Correlation of PEG10 and SOX2 based on TCGA datasets, every dot represented one cancer type.

H Correlation of PEG10 and SOX2 based on CCLE datasets.

I Boxplot illustrated that REACTOME_SIGNALING_BY_NOTCH was enriched in high-PEG10 group based on the TCGA OC datasets.

J Correlation of PEG10 and markers of NOTCH pathway based on TCGA, every dot represents one cancer type.


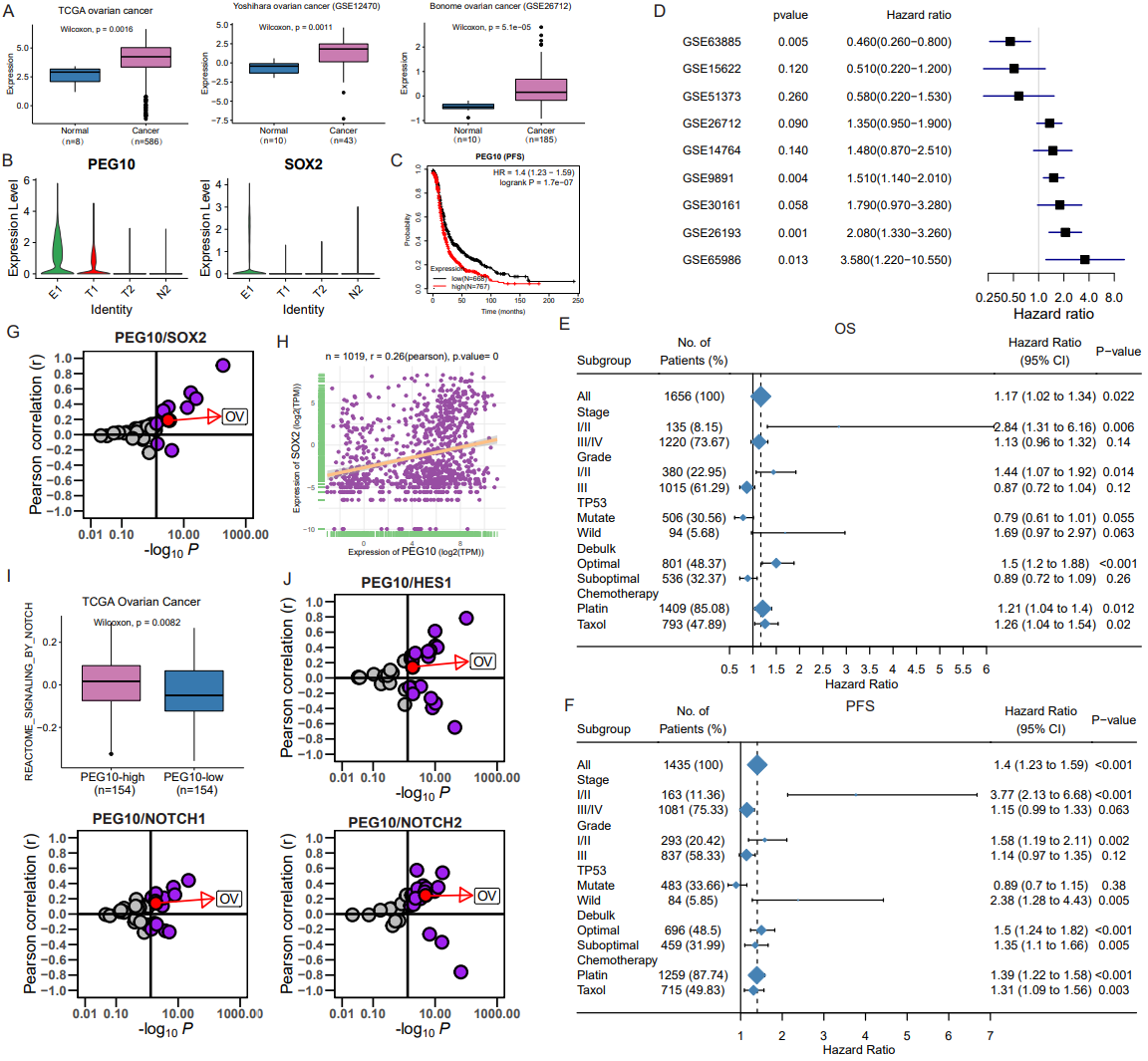


**Fig. S4 Association of 49 genes with OS for 308 patients in TCGA OC cohort.**

A Interaction of 49 genes with OS based on TCGA dataset. The size of each cell represents the survival impact of each gene. The connection of genes represents interactions between both. The thickness of the line indicates the strength of the correlation calculated by Spearman correlation analysis. Red represents positive correlations, and blue represents the negative correlations.

B Heatmap of 49 genes with OS for 308 patients. Each row of the heatmap represents a gene. Each column represents a patient. PT, pharmaceutic treatment.

C Consensus matrices of TCGA OC cohort for k = 2 - 6.


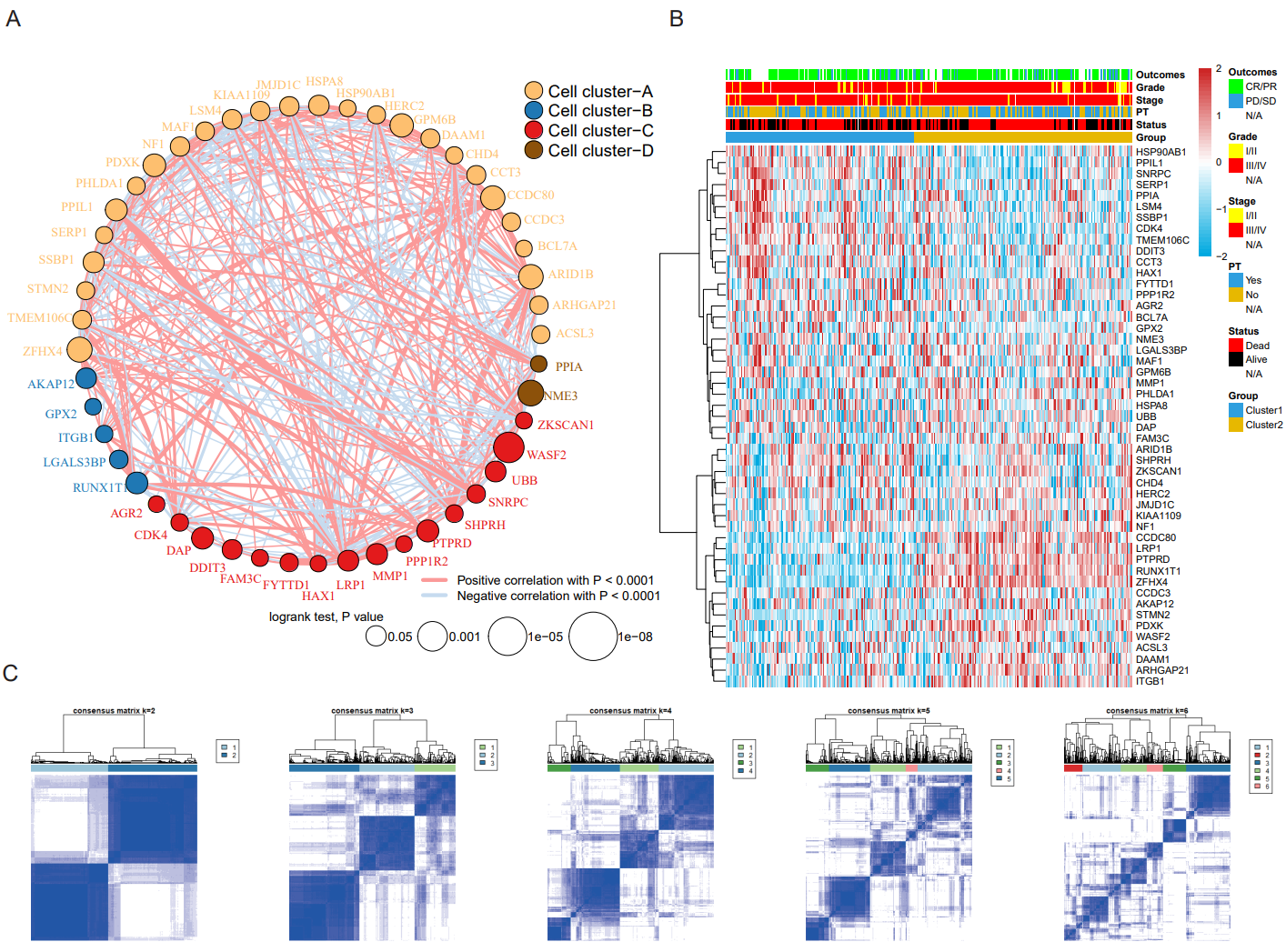


**Fig. S5 Immune molecules in Cluster1 versus Cluster2 in TCGA OC cohort.**

A–C Violin plots displaying the expression of Immune checkpoints (A) Human leukocyte antigens, (B) Immune cells, (C) in Cluster1 and Cluster2. HLA, human leukocyte antigen; DC, dendritic cell; aDC, activated dendritic cell; iDC, immature dendritic cell; pDC, plasmacytoid dendritic cell; NK, natural killer; Th, helper T; Tcm, [central memory T cells](http://www.baidu.com/link?url=PXQdtcLRiGQKr8cZ1CdC6aX-k4xXIBcnRrD3lL_blkU55kSICQc5vQXqEj9wcUoegtASYNlYixOOnzhSmCXqUO2rXYY17VCTow3fZ-mcXqe); Tem, [effector memory T cells](http://www.baidu.com/link?url=PXQdtcLRiGQKr8cZ1CdC6aX-k4xXIBcnRrD3lL_blkU55kSICQc5vQXqEj9wcUoegtASYNlYixOOnzhSmCXqUO2rXYY17VCTow3fZ-mcXqe), TFH, T follicular helper cells; TReg, regulatory cells; Tgd, gamma delta T cell.

D Heatmap illustrating the immune molecules in the two clusters.


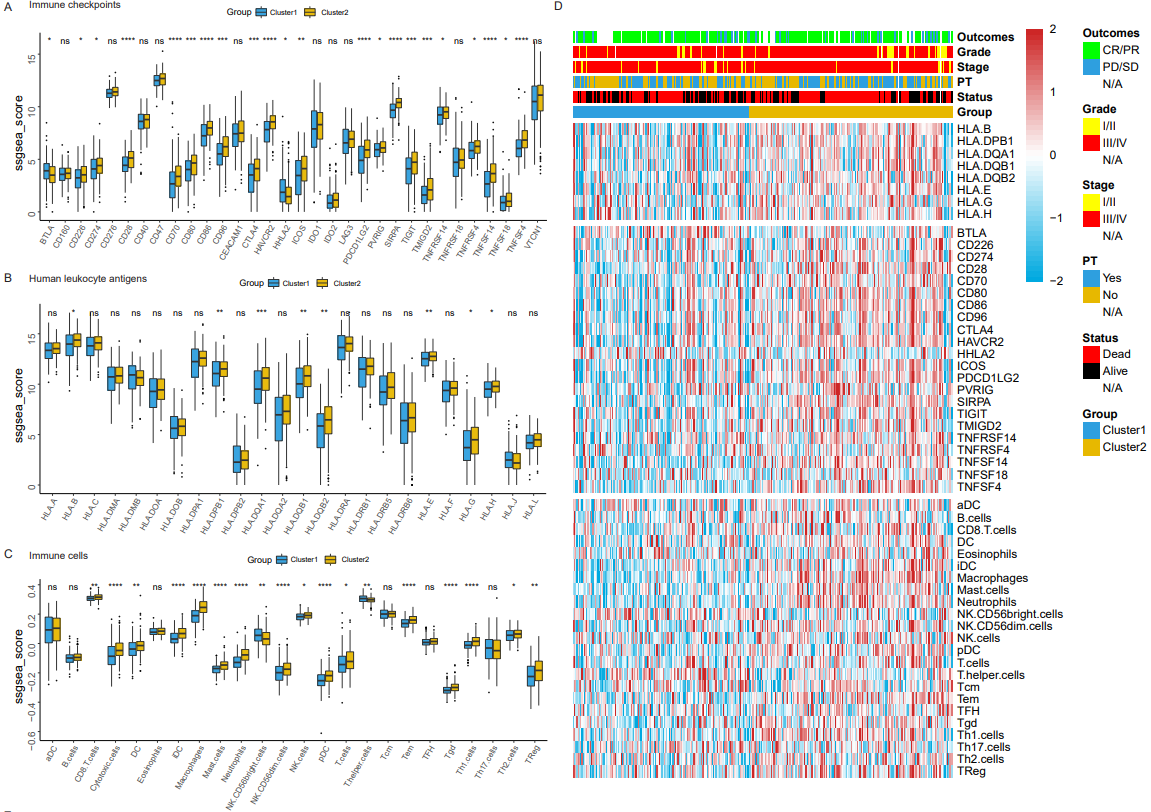


**Fig. S6 Relationships of cytolytic activity (CYT) and immune molecules in TCGA OC cohort.**


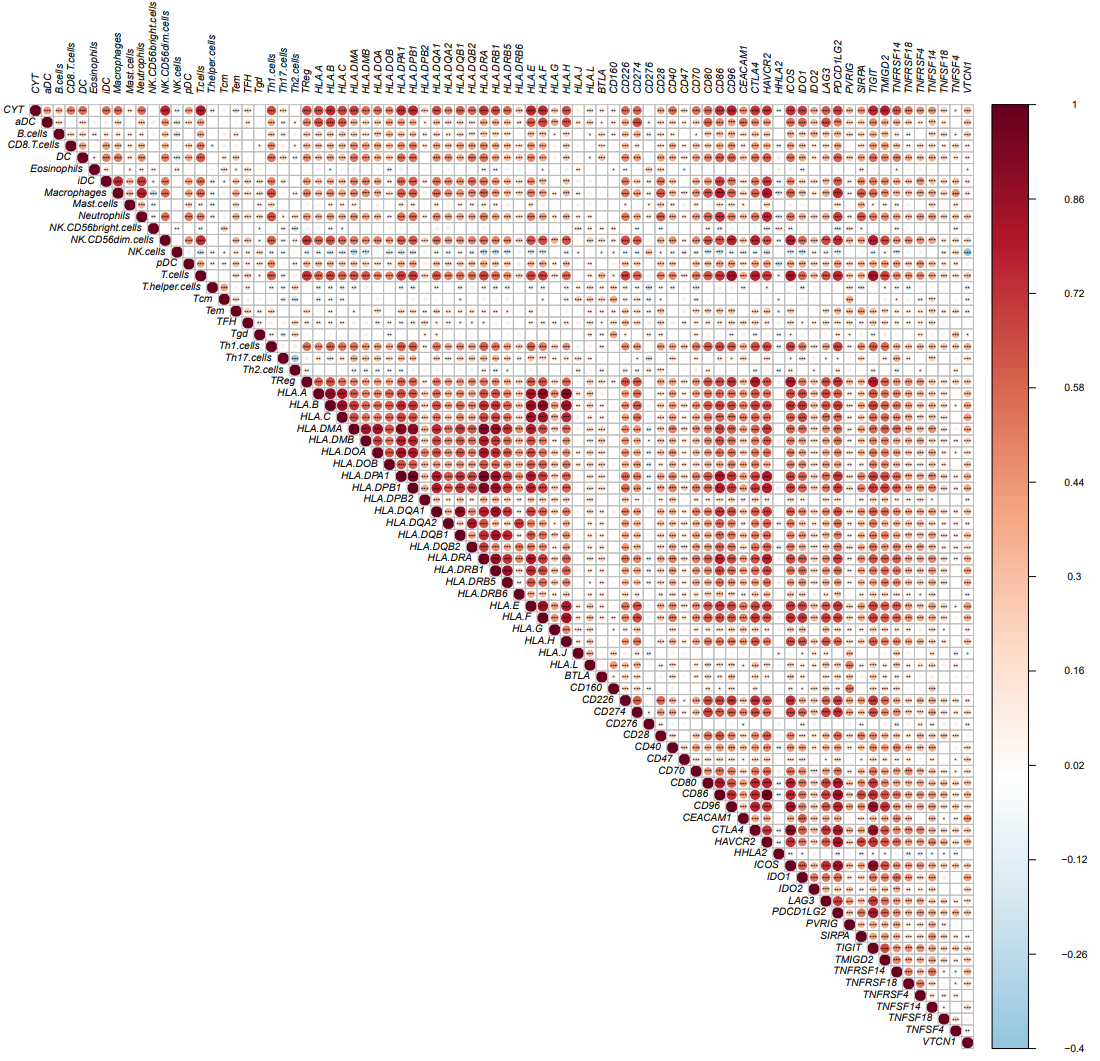


**Fig. S7** **Association of cytolytic activity (CYT) with OS in multiple cancer cohorts.**


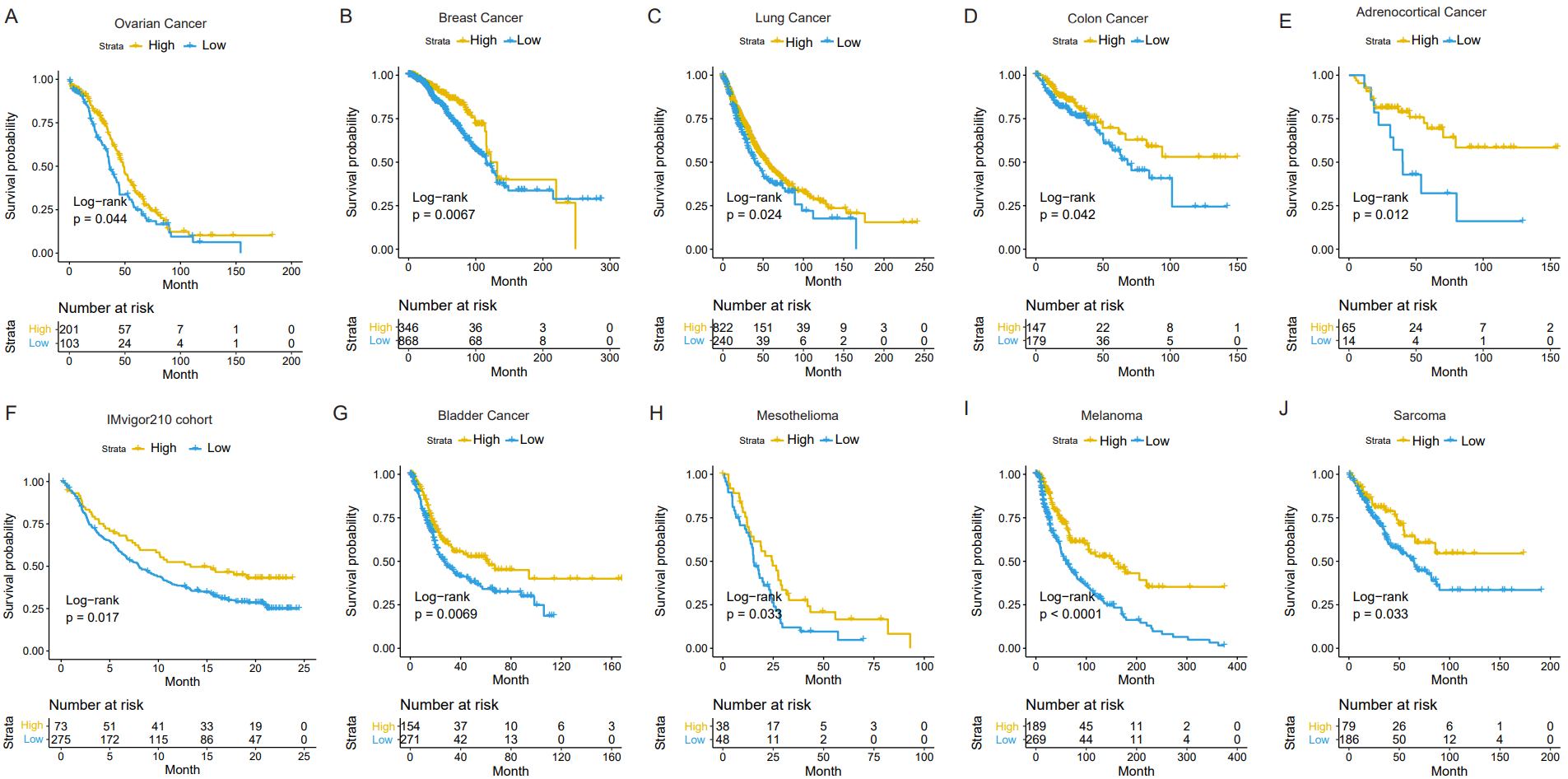


**Fig. S8 Immune molecules of four immune phenotypes in TCGA OC cohort.**

A–C Violin plots displaying the expression of immune checkpoints (A) Human leukocyte antigens and (B) Immune cells (C) in four immune phenotypes.


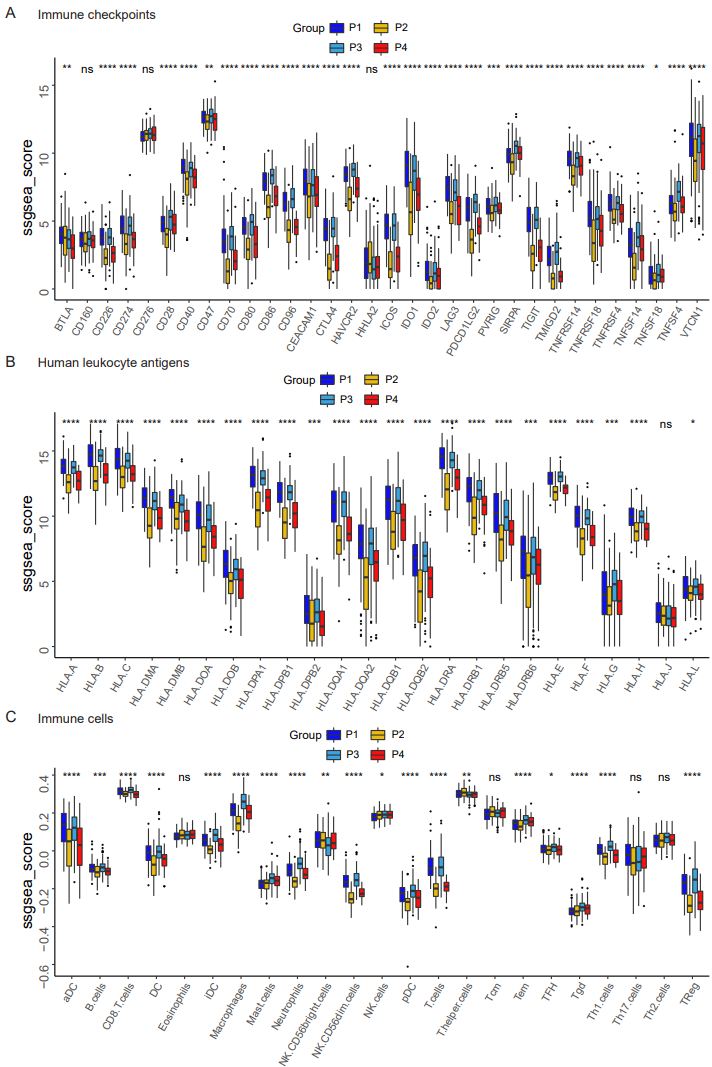


**Fig. S9** **Identification the special gene modules for each phenotype.**

A, B Boxplot showing the different CYT in four phenotypes based on GEO OC meta datasets (A) and IMvigor210 cohort (B).

C Kaplan-Meier curves for patients with four distinct phenotypes in IMvigor210 cohort (Log rank P = 0.0075).

D Relationships of patients’ status, clinical response to anti-PDL1 immunotherapy, and four phenotypes based on IMvigor210 cohort.

E Sample dendrogram and trait heatmap.

F Analysis of the soft thresholding power.

G Dendrogram produced by average linkage hierarchical clustering of the identified co-expression modules.

H Module-trait relationships between the identified modules and four phenotypes.

I Heatmap illustrating the gene patterns of four gene modules.

J–L GO analysis of the P1(J), P2 (K), P3 (L), P4(M).


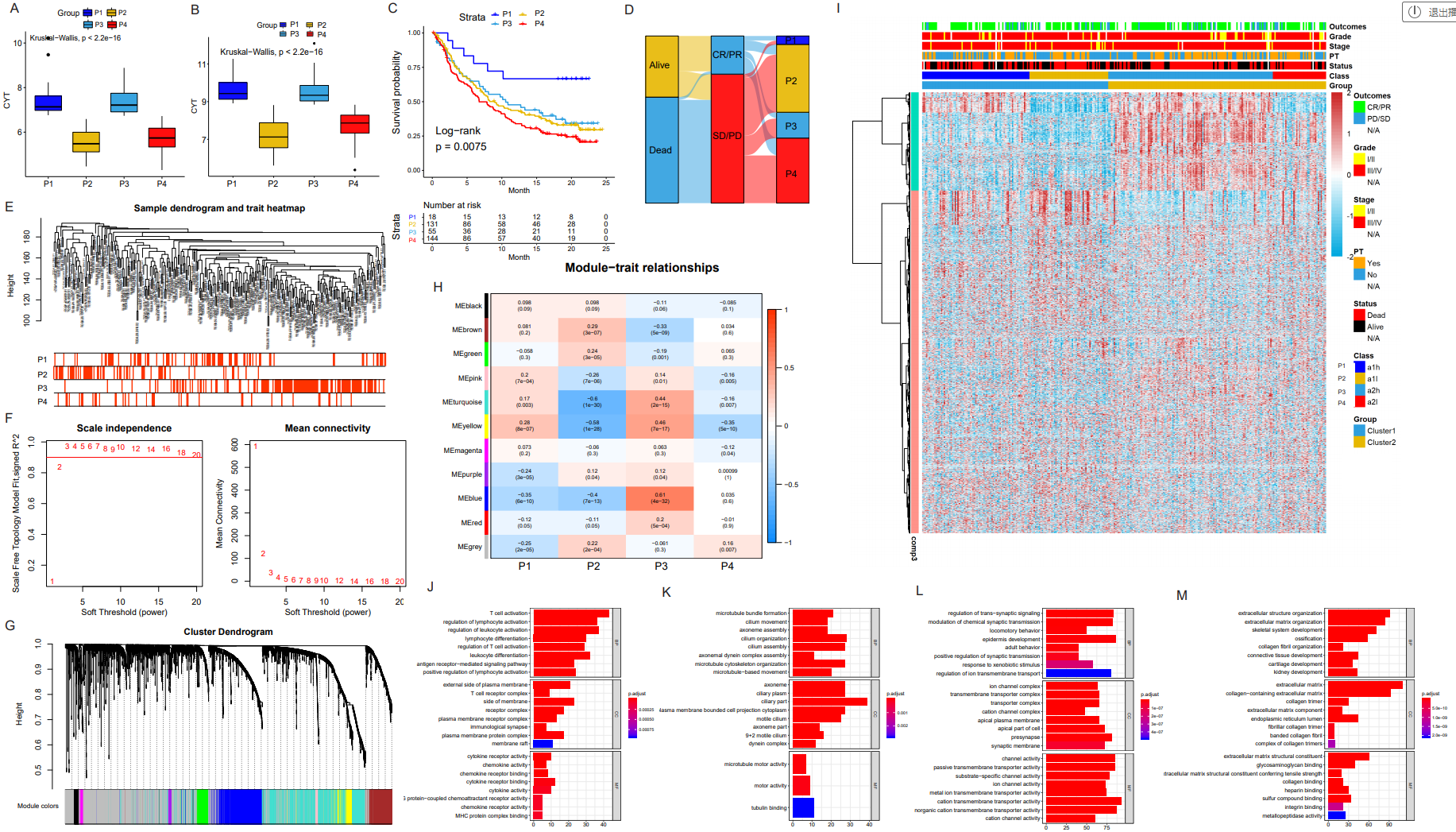


**Table S1. Clinical characteristics of OC patients, control sample and embryo**

| Patient ID | J568541 | J558512 | J558512 | J543993 |
| --- | --- | --- | --- | --- |
| pathologic diagnoses | High-grade serous carcinoma of ovary with omental metastasis， lymph nodes metastasis，appendiceal and gastric surface metastases | Borderline ovarian cancer (Right ovary) | Normal ovary control from Borderline ovarian cancer（Left ovary） | Voluntary abortion，Embryo 9 weeks |
| Age(y/o) | 57 | 26 | 26 | 29 |
| Race | ethnic Han | ethnic Han | ethnic Han | ethnic Han |
| Location | China | China | China | China |
| Stage | IIIC | - | - | - |
| CA199(U/ml) | 7.04 | 59.57 | 59.57 | - |
| CEA(μg/L) | 1.98 | 0 | 0 | - |
| CA125(U/ml) | 272 | 44.3 | 44.3 | - |
| CK7 | positive | - | - | - |
| WT1 | positive | - | - | - |
| PAX8 | positive | - | - | - |
| PR | positive | - | - | - |

**Table S2. List of public datasets used in the study**

**The Cancer Genome Atlas (TCGA) OC datasets**

| Data | Cancer | Samples | Download |
| --- | --- | --- | --- |
| RNA-seq | Ovarian Cancer | 308 | UCSC Xena |
| RNA-seq | Breast Cancer | 1214 | UCSC Xena |
| RNA-seq | Lung Cancer | 1062 | UCSC Xena |
| RNA-seq | Colon Cancer | 326 | UCSC Xena |
| RNA-seq | Adrenocortical Cancer | 79 | UCSC Xena |
| RNA-seq | Bladder Cancer | 425 | UCSC Xena |
| RNA-seq | Mesothelioma | 86 | UCSC Xena |
| RNA-seq | Melanom | 478 | UCSC Xena |
| RNA-seq | Sarcoma | 265 | UCSC Xena |
| RNA-seq | Stomach Cancer | 411 | UCSC Xena |
| RNA-seq | Glioblastoma | 166 | UCSC Xena |
| RNA-seq | Head and Neck Cancer | 565 | UCSC Xena |
| RNA-seq | Cervical Cancer | 307 | UCSC Xena |
| RNA-seq | Pan-Cancer (PANCAN) | 10535 | UCSC Xena |
| RNA-seq | GTEX | 9783 | UCSC Xena |
| Somatic mutation status | Ovarian Cancer | 436 | R package TCGAbiolinks |

**IMvigor210 trial**

| Data | Cancer | Samples | Download |
| --- | --- | --- | --- |
| RNA-seq | Urothelial Cancer | 348 | R package IMvigor210CoreBiologies |

**Kaplan-Meier plotter OC datasets**

| Samples | Survival | Download |  |
| --- | --- | --- | --- |
| 1435 | PFS | http://www.kmplot.com | |
| 1656 | OS | http://www.kmplot.com | |

**The Gene Expression Omnibus (GEO) OC datasets**

| Platform | GSE ID | Samples | Download |
| --- | --- | --- | --- |
| Affymetrix HG-U133 Plus 2.0 (GPL570) | GSE18520 | 53 | GEO database |
| Affymetrix HG-U133 Plus 2.0 (GPL570) | GSE26193 | 107 | GEO database |
| Affymetrix HG-U133 Plus 2.0 (GPL570) | GSE30161 | 58 | GEO database |
| Affymetrix HG-U133 Plus 2.0 (GPL570) | GSE9891 | 285 | GEO database |
| Affymetrix HG-U133 Plus 2.0 (GPL570) | GSE63885 | 101 | GEO database |
| Affymetrix HG-U133 Plus 2.0 (GPL570) | GSE65986 | 55 | GEO database |
| Illumina NextSeq 500 | GSE130000 | 8 | GEO database |

**Oncomine datasets**

| Data | Samples | Download |
| --- | --- | --- |
| TCGA Ovarian cancer | 8 normal samples and 586 tumor samples | http://www.oncomine.org |
| Bonome Ovarian cancer | 10 normal samples and 185 tumor samples | http://www.oncomine.org |
| Yoshihara Ovarian cancer | 10 normal samples and 43 tumor samples | http://www.oncomine.org |

**Table S3. Gene primer sequence**

| Gene symbol | Primer sequence (5'--3') |
| --- | --- |
| PEG10-F | AGCAGTCGGAGGAGAACAAC |
| PEG10-R | CACTGGGCCATGAAAGGAG |
| SOX2-F | GCCGAGTGGAAACTTTTGTCG |
| SOX2-R | GGCAGCGTGTACTTATCCTTCT |
| POU5F1-F | CTGGGTTGATCCTCGGACCT |
| POU5F1-R | CCATCGGAGTTGCTCTCCA |
| NANOG-F | TTTGTGGGCCTGAAGAAAACT |
| NANOG-R | AGGGCTGTCCTGAATAAGCAG |
| NOTCH1-F | GAGGCGTGGCAGACTATGC |
| NOTCH1-R | CTTGTACTCCGTCAGCGTGA |
| NOTCH2-F | CAACCGCAATGGAGGCTATG |
| NOTCH2-R | GCGAAGGCACAATCATCAATGTT |
| JAG1-F | GTCCATGCAGAACGTGAACG |
| JAG1-R | GCGGGACTGATACTCCTTGA |
